# Approaches to Enhance Precise CRISPR/Cas9-Mediated Genome Editing

**DOI:** 10.3390/ijms22168571

**Published:** 2021-08-09

**Authors:** Christopher E. Denes, Alexander J. Cole, Yagiz Alp Aksoy, Geng Li, Graham Gregory Neely, Daniel Hesselson

**Affiliations:** 1 The Dr. John and Anne Chong Lab for Functional Genomics, Charles Perkins Centre and School of Life & Environmental Sciences, The University of Sydney, Sydney, NSW 2006, Australia; christopher.denes@sydney.edu.au (C.E.D.); geng.li@sydney.edu.au (G.L.); 2 Centenary Institute, The University of Sydney, Sydney, NSW 2006, Australia; a.cole@centenary.org.au; 3 Faculty of Medicine and Health, The University of Sydney, Sydney, NSW 2006, Australia; 4 Sydney Medical School, The University of Sydney, Sydney, NSW 2006, Australia; yaks0757@uni.sydney.edu.au; 5 Department of Biomedical Sciences, Faculty of Medicine and Health Sciences, Macquarie University, Sydney, NSW 2113, Australia

**Keywords:** CRISPR/Cas9, genome editing, homology-directed repair, small molecules, engineered Cas9

## Abstract

Modification of the human genome has immense potential for preventing or treating disease. Modern genome editing techniques based on CRISPR/Cas9 show great promise for altering disease-relevant genes. The efficacy of precision editing at CRISPR/Cas9-induced double-strand breaks is dependent on the relative activities of nuclear DNA repair pathways, including the homology-directed repair and error-prone non-homologous end-joining pathways. The competition between multiple DNA repair pathways generates mosaic and/or therapeutically undesirable editing outcomes. Importantly, genetic models have validated key DNA repair pathways as druggable targets for increasing editing efficacy. In this review, we highlight approaches that can be used to achieve the desired genome modification, including the latest progress using small molecule modulators and engineered CRISPR/Cas proteins to enhance precision editing.

## 1. Introduction

### 1.1. Significance and Relevance of CRISPR/Cas9 Technology

Clustered Regularly Interspaced Short Palindromic Repeats (CRISPR) is a gene-editing technology comprising a programmable single guide RNA (sgRNA) that directs a CRISPR-associated protein (e.g., Cas9) to a complementary DNA target sequence to induce single- or double-stranded DNA cleavage [1,2]. Double-stranded breaks (DSBs) are introduced at sites where two critical pre-requisites are met: sequence complementarity between the sgRNA and the target DNA, and the presence of a protospacer adjacent motif (PAM) immediately downstream of the target site [1]. Cas9 cuts both target and non-target DNA strands ~3–4 nucleotides (nt) upstream of the PAM site, producing DSBs that undergo repair by the cellular DNA damage response pathways. The ability to generate targeted DNA cuts provides an entry point for performing a template-directed repair, a long-sought-after goal for treating genetic human diseases. Although several platforms can generate precise mutations, the simple and robust design parameters for CRISPR/Cas9 technology have made it the most widely utilized DNA engineering tool. Potential applications within both laboratory and clinical settings include the rapid generation of cellular and animal models of disease [3], genome-wide functional screening [4], transcriptional modulation [5], live imaging of the cellular genome [6] and gene therapy [7].

### 1.2. Potential Medical Applications of CRISPR/Cas9

The heritable and penetrant genetic basis of diverse human disorders has led researchers to focus on clinical applications for CRISPR/Cas9. Early-stage human trials commenced with ex vivo editing of cells prior to transplantation. One of the first proof-of-principle trials was recently conducted in an HIV-positive patient receiving a bone marrow transplant for lymphoblastic leukemia [8]. CRISPR/Cas9 was successfully employed to knockout CCR5, a critical host cell receptor for HIV entry [9]. CRISPR/Cas9-edited donor cells persisted for at least 19 months after transplantation, without adverse events [8]. Similarly, another proof-of-principle study that achieved multiplexed editing of both the endogenous T cell receptor (TCR) and immune checkpoint PD-1 in chimeric antigen receptor (CAR) T-cells resulted in successful engraftment for at least 9 months without editing-associated clinical toxicities [10]. Recently, the feasibility of treating genetic diseases caused by single-gene mutations was established in a Phase 1 trial for transthyretin amyloidosis, a disease caused by the accumulation of misfolded transthyretin (TTR) [11]. TTR, which is primarily produced in the liver, was targeted with nanoparticle-encapsulated Cas9 mRNA and a single *TTR* guide RNA. Durable knockout was achieved in six patients without any major adverse events [11].

These early trials demonstrate the immense therapeutic potential of the CRISPR/Cas9 gene-editing system. Other studies, however, have revealed significant challenges remain, preventing widespread adoption of precision editing in medicine. Of particular concern is the propensity for generating large deletions; in two recent studies, large regions of the genome were rearranged or deleted in a significant proportion of edited embryos [12,13]. Thus, a major hurdle for medical applications of CRISPR/Cas9 gene-editing lies in improving target editing efficiencies while prohibiting the production of off-target mutations.

### 1.3. NHEJ vs. HDR

**Figure 1:**
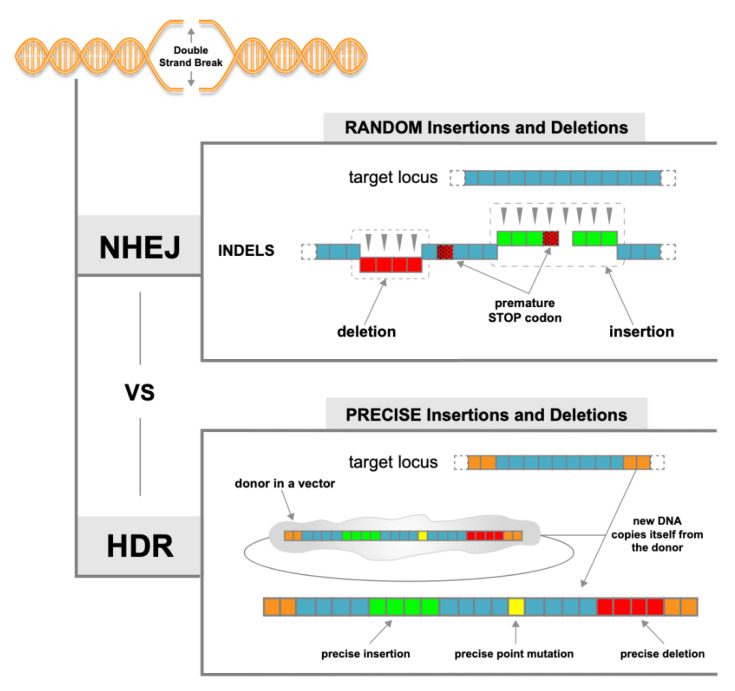
Major DNA repair pathways. Double-stranded breaks are repaired by the error-prone non-homologous end joining (NHEJ) or the precise homology-directed repair (HDR) pathways.

When Cas9 induces DSBs, the endogenous cell-repair machinery attempts to repair the break using one of two major mechanisms: non-homologous end-joining (NHEJ) or homology-directed repair (HDR) (Figure 1; [14]). NHEJ is an error-prone process that is the predominant pathway for repairing DSBs within mammalian cells. A dimeric Ku70/Ku80 complex engages the newly available DNA ends and undergoes a conformational change that protects the free DNA ends, preventing 5′ end resection [14]. Maintenance of minimally processed DNA end structures is required to prevent mutation of the cut sequence during NHEJ-mediated repair [15]. DNA ligase IV re-ligates the cut ends; however, most NHEJ-mediated repair events introduce small non-templated insertions/deletions (indels) that can disrupt gene function [16].

The major therapeutic potential of the CRISPR/Cas9 system lies in the introduction of precise edits at targeted sites. For this reason, HDR is often the desired DNA repair pathway. HDR of DSBs requires 5′ to 3′ end resection, resulting in single-stranded DNA (ssDNA) 3′ overhangs [17]. A repair template carrying a homologous sequence is provided concurrently for editing/replacement of the original DNA sequence [18]. These donor templates can be in the form of ssDNA or double-stranded DNA (dsDNA) [18] and are delivered to the cell as part of the gene-editing process in a variety of forms including plasmids or PCR products. In practice, successful HDR requires DSBs with the correct temporal and spatial coordinates, the availability of a repair template and activated HDR machinery. These requirements render HDR inefficient, especially within somatic cells [19]. Thus, the ubiquitous NHEJ pathway is typically favored by human cells.

### 1.4. Shifting the Balance in Favor of HDR-Mediated DNA Repair

Several methods have been developed to shift the editing balance in favor of HDR. This concept is supported by genetic studies in which *Drosophila* strains lacking DNA ligase IV demonstrate enhanced HDR efficacy [20]. Similarly, the downregulation of NHEJ by siRNA- or shRNA-mediated gene silencing, or gene knockout, indirectly improves HDR efficiency [21,22,23]. Importantly, these pathways are amenable to chemical reprogramming with the aid of small molecule modulators to stimulate HDR, inhibit NHEJ, or achieve both outcomes simultaneously. 

Increased HDR efficiency and/or development of HDR-independent approaches for genome editing are necessary to increase the safety of CRISPR/Cas9 technologies for therapeutic applications. In this review, we discuss recent progress towards enhancing the precision of CRISPR/Cas9-mediated genome editing.

## 2. Small Molecule Modulators

**Figure 2:**
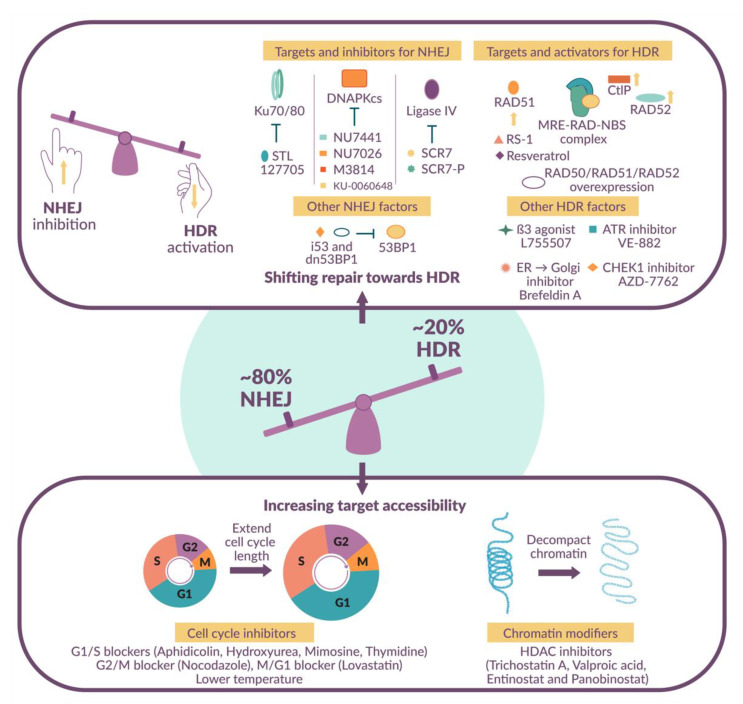
Approaches for enhancing precision editing. Manipulations that favor HDR over NHEJ or which increase the accessibility of the genomic target can increase editing efficacy.

Pharmacological modulation of the DSB repair pathway offers temporal control and reversibility post-editing. A large body of research has focused on small molecule inhibitors of NHEJ, activators of HDR, and cell cycle inhibitors to promote HDR in a gene-editing setting. Indirect methods have focused on inhibiting critical NHEJ factors, such as DNA ligase IV and DNA-dependent protein kinase family members [21,23,24,25], or extending the length of the S and G2 phases of the cell cycle to promote HDR [26]. More direct methods include specifically enhancing the activity of HDR components such as RAD51 [27,28]. Each of these targets influences a different aspect of DNA repair and the simultaneous targeting of multiple factors is emerging as a robust approach to further enhance HDR (Figure 2; [29]).

### 2.1. NHEJ Inhibitors

DNA-dependent protein kinase (DNA-PK) is a master regulator of DNA DSB repair, comprising a catalytic subunit (DNA-PKcs) and the Ku70/Ku80 heterodimer. DNA-PK is responsible for recognizing DSBs, phosphorylating H2A histone family member X (H2AX), and recruiting/activating the repair machinery to induce repair via NHEJ. Numerous studies have demonstrated that inhibiting these DNA-PK proteins can bias the DNA damage response pathway decision towards HDR [30]. Consequently, DNA-PK inhibition was a major target for increasing HDR efficiency. 

Inhibition of DNA-PKcs with the small molecules NU7441 and KU-0060648 reduced the frequency of NHEJ by up to 2-fold and increased HDR up to 4-fold when used in combination with oligonucleotide donor templates [31]. In zebrafish, NU7441 was shown to increase HDR by more than 10-fold [32]. These data are consistent with a similar study in pluripotent stem cells demonstrating another DNA-PKcs inhibitor, NU7026, can increase oligonucleotide donor integration efficiency by up to 1.6-fold [29]. Interestingly, NU7026 was also able to increase Cas12a-targeted nucleotide substitutions. More recently, M3814, a more potent DNA-PKcs inhibitor, produced a 4-fold increase in HDR compared to a 1.7-fold increase with NU7026 [23]. M3814 has enabled simultaneous editing of multiple loci simultaneously [23], and more recently biallelic HDR in murine stem cells [33].

Targeting the Ku70/Ku80 heterodimer has produced mixed results. Suppression of Ku70 and Ku80 using CRISPR significantly increased HDR efficiency [34], consistent with earlier studies of Ku70 and Ku80 shRNA-mediated downregulation [21]. However other studies have shown downregulation of Ku70 had no effect on HDR [22] or decreased both NHEJ and HDR [35]. Recently, a small molecule inhibitor (STL127705) of Ku70/Ku80 heterodimers was developed [36]. Although there are no reports testing the effects of this inhibitor on HDR efficiency, a study testing STL127685 (a 4-fluorophenyl analog of STL127705) showed no effect on CRISPR efficiency [29].

Due to the essential role of DNA ligase IV in ligating DSB ends during NHEJ repair, DNA ligase IV inhibitors were used to inhibit NHEJ and thus promote HDR. SCR7 is the most well-characterized DNA ligase IV inhibitor and was shown to increase HDR activity by up to 19-fold and shift genetic editing events from NHEJ deletions to HDR insertions in a wide range of cell lines including lung, melanoma, breast and colon cancer cell lines, and primary fibroblasts [37,38,39]. In vivo application of SCR7 gave similar results, increasing HDR efficiency in mouse embryos 10-fold [40], and by 46% in rats [41]. Autocyclization of SCR7 results in a more stable cyclized form (SCR7-cyclized), which can then be further oxidized to SCR7-pyrazine; both modified forms of SCR7 are capable of inhibiting NHEJ, although SCR7-pyrazine is less specific [42]. SCR7-pyrazine was demonstrated to increase genetic editing by 50% in MCF7 cells [43]. The efficacy of SCR7-pyrazine in increasing gene knock-in by HDR in *Xenopus* oocytes was less effective, demonstrating a mild 7.4–22% improvement [44]. Overall, the activity of SCR7 appears to be tissue- and organism-specific, with treatments in the embryonic stem cell line H1 [45], the myelogenous leukemia cell line K562 [46], zebrafish embryos [32], and rabbit embryos [47] failing to alter NHEJ:HDR ratios. SCR7 also failed to increase CRISPR/Cas12a-mediated knock-in efficiencies in human pluripotent stem cells [48]. Accordingly, more work is required to define which factors determine how tissue(s) will respond to SCR7 treatment.

Ubiquitylated H2A at DSBs is recognized by 53BP1 to prevent end resection in G1 [49], thereby promoting NHEJ. BRCA1 inhibits 53BP1 function during S phase to promote HDR [50]. Consequently, 53BP1 has become a target for improving HDR. Screening of a ubiquitin variant library identified variant i53 as a significant inhibitor of the interaction between 53BP1 and ubiquitylated histones at DSBs [25]. Co-expression of i53 with either single- or double-stranded donor templates increased HDR efficiency, with double-stranded template co-delivery improving insertion efficiencies up to 2.3-fold [25]. 

### 2.2. HDR Activators

In contrast to inhibiting NHEJ, HDR efficiency can also be increased by directly activating HDR regulators [51]. RAD51 mediates HDR by binding to ssDNA that arises from the end resection of DSB sites. A small-molecule screen identified the compound RS-1 which stimulates RAD51 binding to ssDNA [27] and was later shown to increase HDR insertion efficiencies up to 6-fold and increase knock-in rates using Cas9 nickases (nCas9) [28], both in vitro [28,43] and in vivo [47,52]. However, others failed to see an effect of RS-1 on HDR [46], suggesting that its effect is also cell-type specific. The stilbenoid polyphenol resveratrol, found in the skin of red grapes, was also shown to increase RAD51 expression along with other HDR-associated genes including RAD50, RAD52, BRCA1/2 and RAD51, and to increase CRISPR efficiency by 3-fold [39].

### 2.3. Cell Cycle Inhibitors

As HDR occurs during the S and G2 phases of the cell cycle, several studies have paired cell cycle inhibitors with genetic editing. In one study, a panel of six reversible chemical cell-cycle inhibitors including four G1/S blockers (aphidicolin, hydroxyurea, mimosine, and thymidine), a G2/M blocker (nocodazole) and an M/G1 blocker (lovastatin) were evaluated [26]. To allow for the rapid and synchronized expression of Cas9, cells were nucleofected with a preassembled Cas9 ribonucleoprotein (RNP) complex. All G1/S blockers increased HDR in neonatal fibroblasts, whereas lovastatin had minimal effects. Once again, the effects were cell-type specific with the G2/M blocker nocodazole showing the most significant enhancement of HDR in HEK293T cells. The importance of G2 extension is supported by an independent study showing nocodazole and another G2/M inhibitor (ABT-751) promote HDR in human stem cell lines and enhance CRISPR genome editing [45]. The cell cycle inhibitor XL413 slows the S phase, extending the S/G2/M phases [46]; XL413 increases HDR and oligonucleotide donor integration efficiency by up to 3.5-fold in both cell lines and primary cells [46]. Interestingly, resveratrol was also shown to increase the proportion of cells in the S phase (in addition to its effect on the HDR proteins discussed above) and increase HDR [39]. Furthermore, indirect methods to slow the cell cycle, such as prolonging the cell cycle by incubating zebrafish embryos on ice, increased HDR by 1.5-fold [32]. Together, these data suggest that extending the cell cycle length is critical and that there is flexibility in the specific phase that can be targeted.

### 2.4. Histone Deacetylase Inhibitors

Chromatin compaction is regulated by a diverse range of epigenetic modifications, including histone acetylation and deacetylation, which occurs via writer and eraser enzymes termed HATs (histone acetyltransferases) and HDACs (histone deacetylases). The compact nature of heterochromatin, maintained by the deacetylated status of DNA-wrapped histones, restricts transcription machinery access and impedes gene expression. Histone acetylation by HATs results in decompaction of the heterochromatin structure into euchromatin, a lightly packed chromatin that is amenable to transcription and expression. CRISPR/Cas9 approaches targeting genes within regions of heterochromatin may benefit from decompaction. Accordingly, inhibition of HDAC classes I and II demonstrated significantly increased gene knockout and knock-in rates, while inhibition of other HDAC classes and HATs did not increase efficiencies. The HDACI/II/III inhibitor entinostat increased the gene-editing frequency by 3.7-fold, while the pan-HDAC inhibitor panobinostat increased efficiencies 10.5-fold [53].

The benefits of HDAC inhibition appear to facilitate HDR in various contexts. Trichostatin A, a Class I/II HDAC inhibitor, was shown to increase gene editing efficiency up to 2.2-fold in nCas9 cells. A screen for genes and compounds which increase HDR found that the Class I/II HDAC inhibitor valproic acid (VPA), in combination with RAD51 overexpression, significantly increased biallelic homologous recombination efficiency in human ES/iPS cells [54], consistent with previous studies testing VPA alone [55].

### 2.5. Additional Targets

In addition to cell-cycle inhibitors, HDACs, HDR activators and NHEJ inhibitors, several other factors can increase CRISPR efficiency. DNA polymerase theta (encoded by the *PolQ* gene) acts in parallel to NHEJ at DSBs [56] and promotes microhomology-mediated end-joining (MMEJ) [57]. The synergistic effect of genetic depletion of polymerase theta, combined with M3814 mediated inhibition of DNA-PK enhances HDR [33], suggesting that simultaneous small-molecule inhibition of NHEJ and MMEJ could further bias repair towards HDR. It will be interesting to determine whether a first-in-class polymerase theta inhibitor, novobiocin (which was recently shown to exhibit synthetic lethality with HDR deficiency in tumor cells [58]) phenocopies polymerase theta knockdown in gene editing applications.

A small-molecule screen for compounds capable of enhancing genomic editing on embryonic stem cells by increasing HDR led to the identification of two molecules: L755507 and brefeldin A [59]. L755507 is a β-3 adrenergic receptor agonist, found to increase HDR insertion by 3-fold, while brefeldin A is a fungal metabolite that inhibits protein transport from the ER to the Golgi apparatus and increased insertion efficiency by 2-fold. The L755507 activity was confirmed in fetal porcine cells and shown to decrease NHEJ repair-related gene expression and upregulate HDR-associated genes including RAD51 [39]. 

An unbiased screen to identify molecules that could increase CRISPR/Cas12a-mediated genome editing identified VE-882, an Ataxia-telangiectasia mutated and Rad3-related kinase (ATR) inhibitor and AZD-7762, a checkpoint kinase 1 (CHEK1) inhibitor, as potential targets [48]. Validation of VE-882 and AZD-7762 demonstrated an increase in gene editing efficiency of 5.9-fold and 2.9-fold in human pluripotent stem cells, respectively. In combination, VE-882 and AZD-7762 increased gene editing efficiency 6-fold [48], suggesting that these molecules do not work synergistically.

As small molecules typically have multiple cellular targets at the experimental doses tested, it will be necessary to validate new targets using genetics (knockout or overexpression of the proposed targets) and/or additional small molecules with non-overlapping secondary targets (e.g., chemical structures with distinct pharmacophores).

### 2.6. Targeting Multiple Pathways Simultaneously

Due to the diversity of pathways shown to enhance gene editing efficiency, several studies have tested combinations of inhibitors targeting multiple pathways simultaneously. A recent study defined a combination of four drugs termed “CRISPY”: two drugs known to increase efficiency as single agents (NU7026 and trichostatin A) and two drugs which exhibited limited and inconsistent effects on gene editing efficiency individually (MLN4924 and NSC 15520) [29]. CRISPY increased efficiencies up to 7.2-fold, which was more than triple the efficacy of any of these agents tested alone. The success of this mix was likely a result of its ability to simultaneously target distinct pathways: inhibition of NHEJ by NU7026, decompaction of heterochromatin by the HDAC inhibitor trichostatin A, arrest of the cell cycle at G2/M phase using MLN4924, and inhibiting interactions of the replication protein A complex with NSC 15520, together promoted HDR and inhibited NHEJ activities. In future studies, it will also be important to determine whether combinatorial modulation of multiple cellular processes also increases the frequency of undesirable off-target mutations.

Other studies targeting only two pathways simultaneously have not succeeded in boosting HDR efficiency. Combinatorial treatment of MCF7 cells with SCR7-pyrazine and RS-1 resulted in a small but non-significant increase in gene editing efficacy over each compound alone [43]. In vivo application of SCR7 and RS-1 combination therapy in zebrafish also failed to demonstrate a significant increase over single agents alone [32]. This suggests that dual targeting (inhibiting NHEJ while increasing HDR) may be too restrictive and a broader approach that additionally targets DNA accessibility by manipulating the cell-cycle and chromatin compaction may be most beneficial. 

## 3. Optimized Nucleic Acid Strategies

Optimization of the structure and format of the donor nucleic acid template can also increase HDR efficiency. 

### 3.1. dsDNA vs. ssDNA Templates

Both ssDNA and dsDNA can be utilized as donor templates for HDR [60]. dsDNA templates are widely used due to their low cost and rapid production. These templates can be delivered in the form of linearized plasmid DNA, circular plasmid DNA (which undergoes self-cleavage to linearize within cells) or as a PCR product [61,62,63]. The homology arms required for dsDNA templates are typically relatively long (0.5–2 kb), though homology arms of <100 base pairs (bp) were used successfully [63,64,65]. dsDNA backbones are favorable for long (>1000 bp) templates for whole gene knock-in experiments [63], although size is a limiting factor as knock-in HDR efficiency is positively correlated with dsDNA homology arm length and negatively correlated to insertion size [22]. 

The flexibility associated with in vitro synthesis of ssDNA templates has made them the preferred template for HDR. The ssDNA used in most experiments is considerably shorter than dsDNA templates (at <500 nt), as are the homology arms, at 30–60 nt in length [65,66]. The overall reduced size of ssDNA makes it more suitable for precise editing or short insertion strategies (<100 nt) instead of whole gene knock-ins [67]. The recent advent of technology capable of in vitro generation of gene length ssDNA (>15,000 nt) [68] will likely impact the future use of ssDNA. 

### 3.2. Modified Donor Templates

Various approaches using structural or chemical modifications of the donor template were shown to improve HDR efficiency, including asymmetric donor DNA [69], phosphorothioate-modification [70], chromatin-modification [71] and conjugation of the template with Cas9 [72,73]. However, many of these strategies are still emerging, with continued development likely to result in their substantial refinement. 

#### 3.2.1. Asymmetric Donor DNA

The dissociation of Cas9 from the target DNA sequence after cleavage of the target DNA duplex is asymmetrical, with Cas9 preferentially releasing the 3′ end of the non-target DNA strand [60]. Rational design of asymmetric donor DNA templates complementary to the non-target DNA strand of the DNA duplex was shown to demonstrate higher HDR efficiencies [69].

#### 3.2.2. Phosphorothioate-Modification

Phosphorothioate-modification replaces the phosphodiester bond between sugar moieties of adjacent nucleotides with a phosphorothioate bond by way of a sulfurizing reagent, in which an oxygen atom is replaced by a sulfur. When applied to ssDNA donor templates, this modification alters their chemical properties, stabilizing the ssDNA by reducing extra- and intracellular nuclease degradation and increases their ability to penetrate the plasma membrane of the target cell. Phosphorothioate-modified ssDNA templates enable insertions of >100 nt [69], and display improved HDR efficiencies [70].

#### 3.2.3. Chromatin-Modification

Donor DNA templates are generally delivered for HDR as naked DNA but the delivery of DNA fragments > 50 bp for precise insertion or replacement remains inefficient. Recently, it was demonstrated that histone-wrapped DNA templates that mimic chromatin have demonstrated 2.3–7.4-fold higher HDR efficiencies compared to unwrapped DNA, along with better editing efficiency of chromosome pairs and less cytotoxicity [71]. The optimal configuration of these histone-wrapped DNAs, however, has not been explored.

#### 3.2.4. Conjugation of Template and Cas9

A major constraint of CRISPR-mediated precise genome editing by HDR is the requirement for a high concentration of the donor DNA at the site of Cas9 cleavage. To increase local concentrations and enhance HDR, several studies have focused on recruiting ssDNA templates to the Cas9-gRNA cleavage complex through various conjugations. One such approach used short oligonucleotide adapter-conjugated Cas9 bound to donor templates via base pairing, achieving co-delivery of Cas9 RNP with the template DNA and improving HDR rates [72,73]. Other effective methods have used site-specific covalent conjugation of template DNA to Cas9 by SNAP-tagging [74] or modification of sgRNAs, such that the donor DNA template is linked to the sgRNA [75]. Although it is not clear which approach will be optimal in all situations, template conjugation could be incorporated as part of a multipronged strategy to improve therapeutic editing efficiency.

## 4. Approaches Using Engineered Cas9

### 4.1. Improved CRISPR/Cas9 Systems

**Figure 3:**
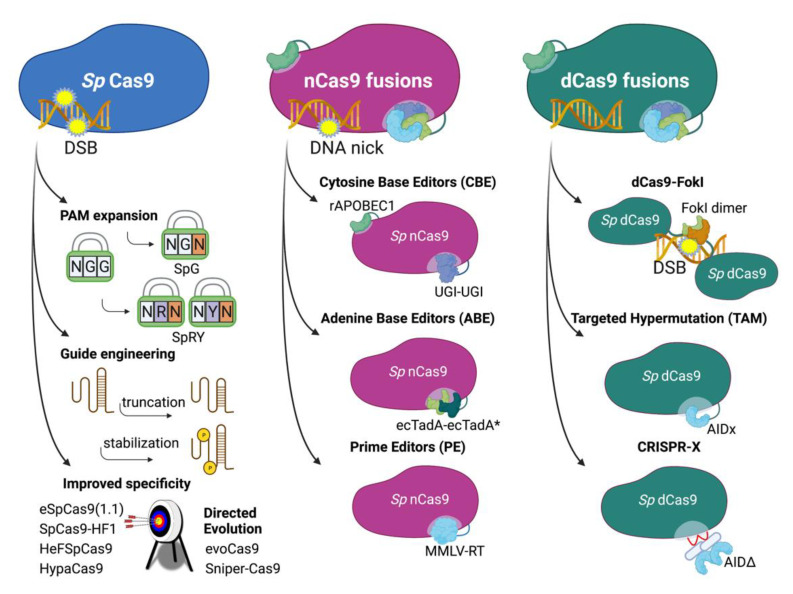
Engineering Cas9 to expand the range of applications and precision of genome editing. *Streptococcus pyogenes* (*Sp*) Cas9 has been modified to reduce target requirements and increase specificity. Partially inactivated (nCas9) and fully inactivated (dCas9) variants have been used to target DNA-modifying activities to specific genomic loci.

Engineering the base CRISPR/Cas9 machinery was performed to increase specificity, activity, efficiency and targeting scope (Figure 3). One of the main challenges faced in genome editing is the availability of PAM sites near targets of interest. A series of Cas9 orthologs were developed that demonstrate expanded PAM site sequence requirements, dramatically improving the editable sequence space of the genome. The first generation SpCas9 recognizes an NGG PAM site [1], but iterations targeting NGAG, NGA and NGCG PAM sites were quickly developed by informed structural design and directed evolution [76] with a broad range of PAM-targeting systems now available [77]. The most universal advance towards improving the targetability of the genome by CRISPR/Cas9 technologies is the development of SpG (a SpCas9 variant with a larger set of NGN PAM sites) and SpRY (a variant that targets NRN PAMs and, less successfully, NYN sites), variants which almost completely eliminate the PAM restrictions of the original system [78]. Alternatively, tethering of Cas9 enzymes to programmable DNA-binding domain proteins (e.g., zinc-finger binding proteins or transcription activator-like effectors (TALEs)) was also shown to vary PAM requirements while decreasing off-target activity [79]. Other Cas enzymes from a variety of species were identified and engineered to further expand the repertoire of editing tools [80,81,82,83,84]. 

Paired nicking is another approach to increasing on-target specificity, requiring the nicking of nearby sites on opposite DNA strands by paired nCas9s [85]. With this strategy, off-target DSB induction rates are reduced as both off-target edit sites would be required to be within close proximity of each other. Similarly, a fusion of a catalytically dead Cas9 (dCas9, a D10A/H840A mutant with inactivated RuvC and HNH domains) to the catalytic domain of the FokI nuclease leads to increased editing specificity, requiring simultaneous recruitment of two separate dCas9-FokI monomers at nearby DNA sites before inducing FokI-mediated DSBs [86,87]. This approach improves target specificity > 140-fold against wild-type Cas9 and at least 4-fold from the paired nickase design (with similar efficiencies).

Furthermore, the use of truncated sgRNAs can also improve editing efficiencies. sgRNAs between 16–19 nt retain the activity of the canonical 20 nt guide sequence though guides < 16 nt in length are inactive [88,89]. An interesting application of this finding is to use < 16 nt sgRNAs to suppress known off-target edits; these “dead RNAs” can be designed to target common off-target sites and block editing when supplied simultaneously with the CRISPR/Cas9 system of choice [90].

The evolution of the Cas enzyme itself has resulted in variants with markedly increased target specificities. The variants eSpCas9(1.1) [91], SpCas9-HF1 [92], HeFSpCas9 [93] and HypaCas9 [94] were generated by rational design and both evoCas9 [95] and Sniper-Cas9 [96] came from directed evolution approaches. The computational high-throughput assessment was able to rank the activity and specificity of such variants and determined that, in general, increased specificity correlates with decreased activity. Further advances are therefore needed to generate highly specific and active variants [97].

Increased HDR frequencies have been demonstrated for fusions of Cas9 with protein elements involved in DNA manipulation, including yeast RAD52 (a DNA repair protein that promotes strand invasion) [98], CtIP (which functions in DNA resection at the early stages of homologous recombination) [99], and peptides derived from chromatin-modulating proteins and complexes [100]. Cas9 enzyme fusions, however, were also developed to simplify the process of genome editing, with donor template-free systems envisioned to remove the inefficient step of HDR.

### 4.2. DNA Base Editors

#### 4.2.1. ABEs and CBEs—Targeted Donor-Free Editing

In 2016, Komor et al. described the first use of CRISPR/Cas9 technology to achieve targeted C→T or G→A transition mutations, a system described as “base editing” [101]. Base editors comprise two critical components: a targetable Cas9 and an ssDNA-modifying enzyme that catalyzes the mutation of targeted nucleotides.

The first-generation cytosine base editor (CBE), BE1, was formed by fusing the rat cytidine deaminase APOBEC1 to the N-terminus of dCas9 [101,102]. APOBEC1 deaminates cytosine to uracil which is base-corrected to thymine by DNA repair mechanisms. Targeting of APOBEC1 to sites of editing interest allows for selective editing of single nucleotides without the known inefficiencies of homology-directed repair or the need for donor template DNA. BE1 was found to have poor editing efficiency [101], but the second-generation editor BE2, which added a uracil DNA glycosylase inhibitor (UGI [103]) to the APOBEC1-dCas9 architecture, increased editing efficiencies to up to 20% in HEK293T and U2OS cells. Subsequent variants have employed improved linkers [104], used a double UGI fusion [105] or incorporated improved APOBEC variants [106] to improve editing rates to up to 80% [107].

With the development of adenine base editors (ABEs) [108], CBEs and ABEs together are capable of targeting almost all of the known pathogenic point mutations in the human genome [101,109]. Similar to BE1, the early editing efficiency of ABEs was poor but directed evolution approaches have improved editing rates to up to 86% [110,111,112].

This rapidly growing field has developed a series of base editors with lower off-target edits [113], tighter editing windows [114], improved editing efficiency [105,115,116,117] and wider PAM targeting [105,118,119,120]. Controlled transversion mutations (C→G) are also now possible with the advent of C→G base editor 1 (CGBE1) and its miniaturized version miniCGBE1 [121]. Simultaneous C and A conversions were also conferred using a single guide base editor (sgBE) system whereby deaminases are recruited to the sgRNA via MS2 interactions in the sgRNA stem-loops, though efficiencies of double edits were low (0.2–8%) with room for improvement [122]. Alternatively, the fusion of cytidine deaminases (codon-optimized PmCDA1 [123] or AID [124]) to both the adenine deaminase TadA and nCas9 produced the Target-ACEmax and ACBE systems which were also demonstrated to achieve simultaneous C→T and A→G substitutions. Importantly, a significant consideration for these single-base donor-free technologies is their impact on RNA deamination [125]. Undesired RNA deamination may produce confounding effects in both research and therapy, particularly when base editors are stably integrated into the system. An alternative approach to the covalent fusion of deaminases to Cas9 enzymes is MagnEdit, a system that achieves cytosine editing by fusing an APOBEC3B interacting protein (hnRNPUL1) to Cas9 to “attract” the APOBEC3B deaminase to targeted sites of DNA [126]. Although improvements to efficiency are still required, the MagnEdit system demonstrated two-fold higher on-target editing efficiency and less target-adjacent edits than an optimized CBE control.

Two adaptations of base editor technology, named CRISPR-STOP and iSTOP, use an ABE to selectively introduce early stop codons in target genes in an attempt to simplify genome-wide knockout screening [127,128]. To facilitate the application of iSTOP, the authors generated an online repository of over 3 million targetable gene co-ordinates for eight eukaryotic species, encompassing up to 99% of the genome [128]. Combining technologies described herein, adapting the PAM specificities of the Cas9 enzymes used in CRISPR-STOP may expand the library of usable sgRNAs and fine-tune the targetable sample space [76,118,129].

Beyond targeting medically relevant point mutations, applications of base editing extend into other fields of research, including directed evolution and drug design. The higher editing efficiencies achieved compared to Cas9-mediated HDR paves the way for significantly more precise genome editing, although reducing off-target effects remains an area of active research [77,107,130].

#### 4.2.2. TAM—Targeted Hypermutation

Targeted activation-induced cytidine deaminase (AID)-mediated mutagenesis (TAM) is a base-editing system capable of hypermutation of targeted cytosines and guanines into any nucleotide without the need for donor template DNA [131]. The TAM system fuses dCas9 to AID, a cytidine deaminase [132,133,134]. Directed by guide RNAs, the fusion of AID-P182X (a truncated AID mutant lacking the nuclear export signal [135]) with dCas9 (dCas9-AIDx) induces random hypermutation of bases clustered around the sgRNA-targeted sequence by cytidine deamination, most frequently upstream of the PAM, with the highest rates at −12 and −16 bp upstream [131]. In vitro, greater than 99.5% of mutations introduced by dCas9-AIDx are nucleotide substitutions, with indels comprising < 0.5%. Alone, AIDx introduces mutations at a preferred (A/T) GCN motif, but the dCas9-AIDx fusion effectively removes any motif requirements for AID-mediated mutagenesis and establishes TAM as a genetic diversification tool for directed evolution. Co-expression of UGI with dCas9-AIDx increased mutation frequencies by up to 5-fold, though substitutions were restricted to C→T or G→A mutations [131].

#### 4.2.3. CRISPR-X—Targeted Hypermutation

Similar to TAM, the CRISPR-X system uses AID to induce hypermutation at cytosines within targeted sites of interest, though the method of AID recruitment differs [136]. In CRISPR-X, dCas9 and AID are not fused but instead interact through an MS2 hairpin/binding protein interaction. sgRNAs containing two MS2 hairpins recruit two MS2 binding proteins, which are fused to a truncated AID lacking its nuclear export signal (described as AIDΔ but which is identical to the AID-P182X in the TAM system; MS2-AIDΔ). Using this AID variant restricts AID localization to the nucleus and was demonstrated to improve on-target mutation in vitro compared to full-length AID and demonstrates the highest mutational frequencies at +12 to +32 bp downstream of the PAM site [136] and an observed mutation rate of ~1/2000 bases [133]. Moreover, a hyperactive AID variant with amplified somatic hypermutation activity increased mutagenesis rates to ~1/1000 bases. Experimentally, the CRISPR-X system was successfully demonstrated for use in the directed evolution of GFP into enhanced GFP (eGFP) as well as in the identification of bortezomib-resistant gene variants [136]. In addition, CRISPR-X was successfully employed to evolve monoclonal human antibodies for increased affinity in a HEK293 cell model [137]. 

### 4.3. Prime Editing—Comprehensive Targeted Base Editing, Insertions and Deletions

An exciting addition to the precision editing toolbox is prime editing, which is capable of facilitating all possible nucleotide conversions as well as targeted insertions and deletions without the need for DSBs or donor template DNA [109]. Directed to target DNA sequences by a prime editing guide RNA (pegRNA), this system utilizes a prime editor (PE; the H840A nCas9 variant fused to the Moloney murine leukemia virus (M-MLV) reverse transcriptase (RT) D200N/L603W/T330P/T306K/W313F variant) to nick the PAM strand of the DNA target. An RNA sequence immediately upstream of the primer-binding site of the pegRNA acts as the template for reverse transcription and contains the edit of interest. Subsequent nicking of the unedited strand can trigger gene conversion using the edited sequence as a template, producing an edited duplex.

The first prime editor (PE1) employed the dCas9 architecture and demonstrated maximal on-target editing efficiencies of 0.7–5.5% with 0.2% indel formation at tested sites [109]. Engineering of the RT variant fused to dCas9 generated PE2 which improved editing efficiency by 1.6- to 5.1-fold above PE1 levels. Optimizing the length of the RT template of the pegRNA, and replacement of dCas9 with nCas9 further increased editing efficiencies, with the resulting editor PE3 capable of 20–50% editing efficiencies (though with increased off-target indel rates of 1–10% dependent upon conditions tested in HEK293T cells). Furthermore, base editing by PE3 demonstrated editing:indel ratios on average 270-fold greater than Cas9-mediated HDR.

Subsequent enhancement of the PE design incorporates enhanced Cas9s with higher on-target specificities (eCas9 and Sniper Cas9 [91,96] to form ePE2 and Sniper-PE2, respectively), prime editors demonstrably less tolerant to mismatches in the sgRNAs used for targeting than canonical PE2 [138]. The genome-wide specificity of PEs can now be quantitatively assessed using nickase-based Digenome-seq (nDigenome-seq) which utilizes whole-genome sequencing to identify PE-induced nicks [138]. 

The functionality of base editors and prime editors are complementary: BEs are useful when bystander edits are acceptable (e.g., for applications such as iSTOP) since they are more efficient and less frequently induce indels, but PEs are advantageous if multiple cytosines/adenines are present within the target site, a PAM site for the BE is unavailable or bystander edits are unacceptable [77].

With the advent of base and prime editing technologies, up to 89% of human pathogenic genetic variants could be targeted for repair, though further improvements to precision are needed [101,109]. As PAM requirements are relaxed, off-target edits are restricted, and on-target activity increases, base and prime editors will likely become core platforms for clinical genome editing.

## 5. Conclusions

The clinical application of CRISPR/Cas-mediated genome editing will require the ability to predictably alter the human genome with minimal off-target events. We anticipate this will be achieved by combining improvements made in the different areas reviewed here such as small molecule gene-editing cocktails with modified ssDNA templates. The effects of specific interventions reviewed here were often cell-type- and tissue-dependent and will therefore need to be specifically re-evaluated in disease-relevant primary tissues. Although base and prime editing approaches are achieving increased flexibility and specificity, manipulation of the cellular and genomic context with small molecules could yield the synergistic improvements in efficacy that are required for therapeutic interventions.

## Data Availability

Not Applicable.

## Abbreviations

ABE, adenine base editor; AID, activation-induced cytosine deaminase; ATR, Ataxia-telangiectasia mutated and Rad3-related kinase; BE, base editor; bp, base pair; CAR, chimeric antigen receptor; Cas9, CRISPR-associated protein; CBE, cytosine base editor; CGBE1, C→G base editor 1; CHEK1, checkpoint kinase 1; CRISPR, Clustered Regularly Interspaced Short Palindromic Repeats; dCas9, catalytically dead Cas9; DNA-PK, DNA-dependent protein kinase; DNA-PKcs, DNA-PK catalytic subunit; DSB, double-stranded breaks; dsDNA, double-stranded DNA; eGFP, enhanced GFP; H2AX, H2A histone family member X; HAT, histone acetyltransferase; HDAC, histone deacetylase; HDR, homology-directed repair; indel, insertion/deletion; M-MLV, Moloney murine leukemia virus; MMEJ, microhomology mediated end joining; nDigenome-seq, nickase-based Digenome-seq; nCas9, Cas9 nickase; NHEJ, non-homologous end joining; nt, nucleotide; PAM, protospacer adjacent motif; PE, prime editor; pegRNA, prime editing guide RNA; RNP, ribonucleoprotein; RT, reverse transcriptase; sgBE, single guide base editor; sgRNA, single guide RNA; shRNA, short hairpin RNA; siRNA, short interfering RNA; ssDNA, single-stranded DNA; TALE, transcription activator-like effector; TAM, Targeted AID-mediated mutagenesis; TCR, T cell receptor; TTR, transthyretin; UGI, uracil glycosylase inhibitor; VPA, valproic acid
